# Psychological Therapies for Auditory Hallucinations (Voices): Current Status and Key Directions for Future Research

**DOI:** 10.1093/schbul/sbu037

**Published:** 2014-06-13

**Authors:** Neil Thomas, Mark Hayward, Emmanuelle Peters, Mark van der Gaag, Richard P. Bentall, Jack Jenner, Clara Strauss, Iris E. Sommer, Louise C. Johns, Filippo Varese, José Manuel García-Montes, Flavie Waters, Guy Dodgson, Simon McCarthy-Jones

**Affiliations:** ^1^Brain and Psychological Sciences Research Centre, Swinburne University, Melbourne, Australia;; ^2^Monash Alfred Psychiatry Research Centre, The Alfred, Melbourne, Australia;; ^3^School of Psychology, University of Sussex, Brighton, UK;; ^4^Research & Development Department, Sussex Partnership NHS Foundation Trust, Brighton, UK;; ^5^Institute of Psychiatry, King’s College London, Department of Psychology, London, UK;; ^6^National Institute for Health Research (NIHR) Biomedical Research Centre for Mental Health at South London and Maudsley NHS Foundation Trust (SLaM), London, UK;; ^7^Psychological Interventions Clinic for Outpatients With Psychosis (PICuP),SLaM, London, UK;; ^8^VU University and EMGO+ Institute for Health and Care Research, VU University, Amsterdam, The Netherlands;; ^9^Parnassia Psychiatric Institute, The Hague, The Netherlands;; ^10^School of Psychological Sciences, University of Liverpool, Liverpool, UK;; ^11^Jenner Consult, AUDITO, Groningen, The Netherlands;; ^12^Department of Psychiatry, University Medical Center Utrecht, Utrecht, The Netherlands;; ^13^School of Psychological Sciences, University of Manchester, Manchester, UK;; ^14^Department of Psychology, University of Almería, Almería, Spain;; ^15^School of Psychiatry and Clinical Neurosciences, University of Western Australia, Perth, Australia;; ^16^Clinical Research Centre, North Metro Health Service Mental Health,Perth,Australia;; ^17^Early Intervention in Psychosis, Greenacre Centre, Ashington, UK;; ^18^ARC Centre of Excellence in Cognition and Its Disorders, Department of Cognitive Science, Macquarie University, Sydney, Australia;; ^19^Department of Psychology, Durham University, Durham, UK

**Keywords:** auditory hallucinations, psychosocial intervention, psychological therapy, cognitive behavioral therapy, psychosis

## Abstract

This report from the International Consortium on Hallucinations Research considers the current status and future directions in research on psychological therapies targeting auditory hallucinations (hearing voices). Therapy approaches have evolved from behavioral and coping-focused interventions, through formulation-driven interventions using methods from cognitive therapy, to a number of contemporary developments. Recent developments include the application of acceptance- and mindfulness-based approaches, and consolidation of methods for working with connections between voices and views of self, others, relationships and personal history. In this article, we discuss the development of therapies for voices and review the empirical findings. This review shows that psychological therapies are broadly effective for people with positive symptoms, but that more research is required to understand the specific application of therapies to voices. Six key research directions are identified: (1) moving beyond the focus on overall efficacy to understand specific therapeutic processes targeting voices, (2) better targeting psychological processes associated with voices such as trauma, cognitive mechanisms, and personal recovery, (3) more focused measurement of the intended outcomes of therapy, (4) understanding individual differences among voice hearers, (5) extending beyond a focus on voices and schizophrenia into other populations and sensory modalities, and (6) shaping interventions for service implementation.

The International Consortium for Hallucinations Research (ICHR) was set up to promote international collaborations on key areas of research related to auditory hallucinations.^1,2^ This article reports on the findings of a working group reviewing psychological therapies for hallucinations to identify key directions in future research. Psychological therapies are widely recommended in clinical practice guidelines as part of the treatment for people who experience psychotic phenomena, particularly for those with medication-refractory psychotic experiences.^3,4^ While previous reviews have considered psychological therapies for psychosis broadly, including recent reviews of practice,^5^ outcomes,^6,7^ new developments,^8^ and implementation issues,^9^ no recent reviews have focused on therapies for hallucinations. Yet, hallucinations, particularly in the form of hearing voices, are a frequent source of distress and interference with functioning, resulting in them being a major target of psychological therapies for psychosis. This article considers issues specific to psychological interventions for hallucinations, with a focus on the phenomenon of hearing voices: hallucinatory experiences involving hearing speech, often with the impression of this being generated by another identity.^10^ Although a broad spectrum of hallucinatory phenomena arise across a range of populations (including nonclinical), we have focused on hearing voices because, as well as this being the most frequent hallucinatory phenomenon encountered in psychotic disorders, voices have been the usual focus of therapies for hallucinations described in the literature to date. This review considers current approaches to research on therapies for voices, limitations in existing research in informing the outcomes of therapy and specific therapy processes, and sets out priorities for ongoing research in this area.

## The Development of Psychological Therapies for Voices

The development of psychological therapies for voices has primarily involved the application of behavioral and cognitive-behavioral methods with persons with psychotic disorders. Early studies adopted behavioral approaches, based upon addressing hypothesized antecedents and reinforcers of voices. These studies examined a range of specific interventions such as relaxation training, graded exposure to voice triggers, manipulation of environmental contingencies for behavioral responding to voices, and even aversion therapy.^11^ Other studies, spurred by research into coping in psychosis, examined training in specific coping methods, in particular manipulation of sensory input using ear plugs or music on headphones and use of distraction techniques, with some evidence of effectiveness (see review by Farhall et al^12^).

Integrating a number of these methods into a functional analysis-based approach, the development of coping strategy enhancement (CSE)^13^ in the early 1990s provided a precursor to formulation-based cognitive-behavioral interventions used today. In CSE, a detailed assessment of modulating factors and responses to voices is used to inform individually tailored modifications to the person’s chosen coping methods. In 2 trials of people with schizophrenia with hallucinations or delusions, CSE resulted in reduced ratings of symptom severity, compared with both treatment as usual (TAU) and supportive counseling.^13,14^

Through the 1990s and 2000s, these behavioral methods were expanded on by the application of cognitive models^15–17^ to psychosis, giving rise to a number of therapy methods collectively referred to as cognitive behavioral therapy for psychosis (CBTp). Usually presented for the range of difficulties associated with psychosis, rather than specifically for voices, CBTp broadly involves working at the meaning level: assisting the person to develop an adaptive understanding of their psychotic experiences, combined with targeted reframing of appraisals linked with distress or interference with functioning.^18^ The model of Chadwick and Birchwood^19^ was particularly influential in the development of a cognitive understanding of voices. Rather than voices always being problematic, this model suggests that beliefs held about the identity, power and intent of voices, and degree of control over the experience, predict distress, depression, and problematic responses to voices such as compliance with command hallucinations. In turn, these beliefs, and the person’s relationship with their voices, may be related to broader schemas about self and others, and to one’s position in the social world.^20^ This model is supported by consistent findings of relationships between appraisals of voices and indices of distress and responses to voices.^21^

In practice, CBT for voices is characterized by an emphasis on engagement and the therapeutic relationship; on an individualized formulation approach, with an emphasis on making sense of voices within a developmental and often interpersonal framework, and aiming for meaningful change within the context of valued goals; on the integration between emotional and psychosis processes; and on changing people’s relationships with their voices.^18^ In addition, beliefs about the power of voices, and compliance behaviors, can be targeted specifically, using methods such as Socratic questioning and behavioral experiments designed to test alternative explanations.

Beyond these specific methods used within individual therapy, it is of course important that interventions for psychosis also consider the context of the person’s family and broader social network. Family and social environment have an important role in relation to mental health, with the understanding that a well-functioning support network equipped with knowledge and coping skills has a significant impact on the recovery, stability, and well-being of people with psychosis,^22^ as well as being predictive of better outcome in therapy.^23^ A comprehensive intervention approach targeting this is hallucination-focused integrative therapy (HIT),^24^ which integrates coping enhancement and CBTp with a variety of motivational strategies, behavioral reinforcement, family therapy, rehabilitation, and crisis management. In this way, HIT promotes key principles of managing voices across a range of contexts, promoting an environment which supports behavior change in the individual, maximizing client adherence and motivation, and promoting generalization within the person’s day-to-day life.

## Empirical Findings for Psychological Interventions for Voices

The effects of psychological therapies for voices have most frequently been evaluated by examining the efficacy of CBTp, as an adjunct to routine care (including antipsychotic medication), on the overall severity of positive symptoms (hallucinations and delusions combined). Despite symptom severity providing quite an indirect index of adaptation to psychosis, and differences in trials included, meta-analyses of randomized controlled trials (RCTs) are consistent in demonstrating evidence for beneficial but modest effects of CBTp on measures of positive symptoms (posttreatment between-group effect sizes ranging from 0.25 to 0.47).^6,25,26^ Methodological differences in trials, such as blinding, may moderate positive symptom effect sizes.^6,26^ There may also be smaller magnitude effects when compared with control therapies as opposed to TAU^26^ (although these effect sizes did not significantly differ in a recent meta-analysis),^6^ but it is likely that control therapies contain more therapeutic elements than a true placebo^27^ and a recent large meta-analysis indicated advantages of CBTp over any active control condition.^7^

However, compared with the large number of studies examining the effects of cognitive and behavioral therapies on the broad and indirect index of positive symptom severity, there is much less direct evidence regarding effects on hearing voices as a specific phenomenon, or the specific elements of therapy that contribute to outcome. There have been few trials focusing specifically on voices, with most studies combining participants with hallucinations and delusions, resulting in sample heterogeneity and a variable focus on voices during therapy. Even though some of these RCTs have added voices measures as a secondary outcome, because voices are only one of a number of possible issues addressed in therapy, smaller overall effects are likely to be observed than if voices were a focus. Indeed the proportion of time spent addressing voices in CBTp trials can be small.^28^ Furthermore, while some individual trials that have included voices measures have observed improvements,^29,30^ including a recent trial with people not taking antipsychotic mediation,^31^ most have been insufficiently powered to be conclusive, due to their small sample sizes^32–34^ or delivery of therapy during acute relapse when substantial reductions in symptoms arise from routine care alone.^23,35^ Nonetheless, a recent meta-analysis^6^ (although limited by combining trials delivered during chronic and acute psychosis, and including trials not directly targeting positive symptoms), observed a significant effect of CBTp on posttreatment voice severity vs any control (Hedge’s *g* = 0.34, 15 studies). Similar findings were observed in a further forthcoming meta-analysis of 15 studies using voice severity as an outcome, which additionally observed that hallucinations effect sizes were not reduced in comparisons with active treatments only, or when unblinded studies were removed (Van der Gaag, personal communication).

In treatment studies specifically focused on voices, thereby reducing sample heterogeneity, there appears to be evidence of improvements on a number of indices of voices (overall severity, voice-related distress, disability, compliance, and voice frequency) in pre- to posttherapy comparisons of CBT-based interventions,^36,37^ including in group^38–42^ and self-paced web-based^43^ formats. Among the small number of RCTs conducted, advantages vs TAU on compliance with harmful command hallucinations have been observed^44^ using a specific command hallucinations protocol based upon the Chadwick and Birchwood^19^ model. Importantly, this trial observed some of the largest effect sizes of all CBT trials with psychosis, suggesting that the use of focused protocols with clearly defined populations and outcomes can produce substantial effects. By contrast, a small RCT comparing a combination of this protocol and acceptance and commitment therapy methods with a befriending-based control therapy had inconclusive findings in a sample with a low base rate of compliance.^45^ However, a large multicenter trial of the cognitive therapy for command hallucinations protocol^46^ is about to report findings of reduced compliance and perceived voice power compared with TAU (Birchwood, personal communication).

Using the comprehensive approach of HIT, improvements have been observed on voice-related distress, overall psychotic symptoms, depression, social functioning, and quality of life,^24,47^ including gains which are maintained at follow-up.^48^ This suggests that integrated intervention can produce broad and persisting benefits. On the other hand, randomized trials of group-based CBT for voices have had disappointing posttherapy advantages over control on voice measures, but have found broader benefits such as improvements in social functioning.^49,50^

## Recent Developments in Psychological Therapies for Voices

Since the early focus of psychological therapies on enhancing coping and reframing beliefs about voices, a number of ongoing developments in therapeutic approaches for voices have emerged. Many of these developments can also be placed under a broad CBT umbrella, but also reflect additional influences.

In line with a general trend in psychological therapies, one major area of development has been the application of acceptance and mindfulness-based approaches to voices. These include mindfulness training and acceptance and commitment therapy (ACT).^51–53^ These interventions focus less on belief change and more on changing the relationship with internal experiences such as hearing voices. A decentered relationship with such experiences, involves an awareness of experiences while maintaining some distance and disidentification from them.^54^ While CBTp helps people to do this as an implicit goal (by observing voices, thoughts, feelings, and behaviors and developing alternative appraisals of the voices), mindfulness and ACT promote this directly, through practising skills in noticing voices, thoughts and feelings as passing events.^55^ This may lead to changes such as reduced believability of voices.^56^ While there is still insufficient data from RCTs on posttherapy between group differences, a recent meta-analysis of acceptance and mindfulness-based therapies for psychosis has found improvements from pre- to posttherapy on measures of positive and negative symptoms, affective symptoms and on measures of quality of life and functioning.^57^

Another trend has been further consolidation of methods for working with voices within the broader context of one’s view of self, relationship with others, and self-narratives that include one’s life experiences. Many voice hearers, especially those who have suffered traumatic events, can experience negative views of self and may additionally see others as potentially dangerous and critical.^58^ These views can parallel voice content, making the voice hearer particularly vulnerable to derogatory and threatening content of voices. In addition to the use of traditional cognitive therapy methods, contemporary approaches including competitive memory training (COMET)^59^ and compassionate mind training (CMT)^60^ have been used to promote greater resilience to critical commenting voices. COMET involves strengthening positive memories that are inconsistent with critical voice content by rehearsing them using imagery. An initial RCT of COMET with voice hearers found reductions in depression which were mediated by changes in perceived voice power and voice acceptance.^59^ CMT involves practising exercises which promote self-compassion and compassion toward others, aiming to activate brain systems involved in social and self-soothing believed to modulate threat systems active when experiencing hostile voices. Other approaches have focused on incorporating aspects of interpersonal relating, past relationships, and attachment into therapy.^61^ A recent innovative approach has been the development of computer-generated avatars that enable the therapist to role-play the voice to aid the person in practising different responses to the experience.^62^ A pilot RCT of this therapy showed reduced overall voice severity and the perceived power and malevolence of voices relative to routine care. Meanwhile, therapy developments stemming from the hearing voices movement, which has long emphasized the value of seeing voices as meaningful in the context of past life experiences (Corstens et al, this issue), include systematic enquiry about the relationship between voices and past experience,^63^ and the therapist directly addressing and questioning voices via the hearer in therapy.^64^

Current trials for people with distressing voices that the authors are aware of include RCTs of mindfulness-based CBT groups (ISRCTN74054823); avatar therapy (ISRCTN65314790); an intervention focusing on relational aspects of voices (ISRCTN44114663); and a peer-delivered intervention focusing on developing shared understanding of voice experience (ACTRN12612000974808). Additionally, large RCTs of CBTp (ISRCTN29242879), ACT for persisting psychosis (ACTRN12608000210370), and trauma-focused intervention in psychosis (ISRCTN79584912) are about to report results including impact on voices.

## Key Research Issues for the Ongoing Development of Psychological Therapies

The above review, and discussion between members of this ICHR working group identified 6 issues that are critical for the ongoing research into psychological interventions for voices.

### Beyond Overall Efficacy to Understanding Therapeutic Processes

The first issue is that the efficacy trials conducted to date and reviewed in recent meta-analyses, appear to have been quite limited in informing the specifics of how therapy should be applied to voices. The typical RCT design has involved recruiting a group of participants experiencing a broad range of psychotic experiences, delivering an individualized (and usually variable) therapy based on a broad range of behavioral and/or cognitive principles, and examining outcomes using broad indices of mental state, such as overall positive symptoms. There has also been a focus on establishing efficacy rather than on measuring the purported mechanisms of change (eg, beliefs about voice omnipotence and malevolence). While such trials were necessary to develop an evidence base for inclusion in national service delivery guidelines, relatively little information about the processes of therapy has been revealed. Similar conclusions have been reached in the (psychological interventions for) delusions literature.^65,66^

Probably the most informative trial so far conducted has been the work on cognitive therapy for command hallucinations, which has shown the benefit of specific model development, and which successfully combined measurement of process and a targeted outcome.^44,46^ Similarly, some of the recent trials, such as those of COMET,^59^ have been beneficial in providing data on specific therapeutic methods. This targeted approach, with highly focused interventions for specific candidate processes, has recently been fruitful in informing intervention with delusions. For example, focused research designs have been useful in identifying both worry and insomnia as variables associated with paranoia, which can be effectively targeted by brief discrete interventions to produce reductions in paranoia.^65^ At this stage in the development of therapies for voices, there is perhaps greater need to identify key mechanisms of therapeutic change than to continue conducting broad efficacy studies.

Related to this point, while it is useful to see developments that increase the technical repertoire for addressing voices, the recent wave of new therapy approaches for voices presents challenges in conceptualizing how these approaches fit together. Developments include methods explicitly framed as elements that might be incorporated within a broader CBT model (eg, COMET, imagery rescripting), and approaches often presented as distinctive therapy modalities (eg, ACT, CMT). Inherent in this diversification is some risk that it may not be feasible for practitioners to develop skills in all methods, and this may give rise to levels of skill in, or degree of allegiance to, particular therapy approaches informing delivery to a greater extent than client need. In addition, the associated brand naming of therapy paradigms may draw research activity toward RCTs trailing yet more broad therapy approaches for broad populations, as opposed to research targeted at understanding processes. Potentially this may generate a culture of contrasting evidence for the efficacy of one therapy variant against another, even though the overall approaches share more common features than they have differences.

This highlights a need to balance the innovation associated with the diversification of therapy developments with potential to integrate them into common themes, or potentially a model of adaptation to hearing voices that cuts across therapy modalities. To this end, table 1 outlines some of the key therapeutic targets which have been described in the literature and current research evidence relating to them. These range from transdiagnostic processes targeted in the context of psychosis (eg, enhancing coping, self-esteem, and compassion), through processes particularly associated with psychosis (eg, understanding psychotic experiences as mental phenomena), to processes more specific to voice hearing (eg, interpersonal relationship between person and voice). Among these, it is apparent that there are particular areas which form routine parts of therapeutic practice (eg, development of an understanding of psychotic experiences) for which the basis has yet to be examined empirically.

**Table 1.** Key Processes Targeted by Psychological Intervention for Voices

Therapy Target	Main Therapeutic Methods Targeting	Key Research Findings
Range and effectiveness of coping strategies	Coping strategy enhancement,^ 13 ^ and discussion of coping in voices groups^ 38,40,50 ^	Evidence for impacts of various coping methods on voices, and that having a greater range of coping strategies being helpful.^ 9 ^ RCT conducted with coping enhancement as a key focus had strong benefits over supportive counseling and TAU.^ 14 ^
Understanding of voices as a mental phenomenon	Normalizing explanations of voices; monitoring and exploring voice phenomenology and continuities with cognition; and formulation within vulnerability stress and biopsychosocial models are methods used in CBTp.^ 18 ^	Not yet established whether an improved understanding of voices (or belief that voices are mentally generated) is predictive of better adaptation. Although a key element of CBT-based approaches, there has been limited specific examination, although a focusing intervention with particular emphasis on this did not find advantages over a distraction-based intervention in a small RCT.^ 36 ^
Perceived power of voices and subjective control over voice experience	Primary focus of cognitive therapy for voices,^ 19 ^ particularly command hallucinations.^ 44 ^	Well-established relationship between these dimensions and emotional impact of voices.^ 21 ^ RCT evidence for therapy targeting this having beneficial impacts on compliance with harmful commands.^ 44 ^
Ability to disengage and decenter from voice experience	Most directly focused on in acceptance and mindfulness-based approaches.^ 67,68 ^ May also arise from cognitive restructuring of content in CBT,^ 69 ^ and addressing beliefs which promote engagement with voices.^ 70 ^	Mindfulness and acceptance are correlated with indices of better adaptation to voices.^ 71,72 ^ Meta-analysis of RCTs of acceptance and mindfulness-based approaches for psychosis showed beneficial general pre- to posteffects, but as yet inconclusive between group or voice-specific findings,^ 57 ^ although large trials of ACT and mindfulness groups pending completion. No focused examination of effects of cognitive therapy targeting voice content.
Understanding of voice content in context of broader life experiences and representations of self and others	Target of broad CBTp model^ 18 ^ and of approaches stemming from the Hearing Voices Network.^ 63,64 ^	Evidence of meaningful patterns being identifiable,^ 73,74 ^ but uncertain whether a greater understanding of this predicts better adaptation. Often included in CBTp trials but not yet examined as a specific component of therapy. Pilot RCT of peer-delivered intervention with particular focus on this due for completion in 2014.
Self-esteem and self-compassion	Reinforcement of memories supporting positive self-image competing with critical voice content through imagery and rehearsal described in COMET.^ 59 ^ Use of imagery and mindfulness to develop self-compassion described in CMT.^ 60 ^	RCT evidence that COMET reduces depression in people with critical voices, mediated by changes in voice acceptance and power, but no change in voice-related distress.^ 59 ^ CMT examined in relation to voices in case series only to date.^ 60 ^
Specific traumatic memories and imagery associated with voices	Imagery rescripting,^ 75 ^ prolonged exposure,^ 76 ^ and eye movement desensitization and reprogramming^ 77 ^ have been described in case series.	Although an association between trauma and voices has been established,^ 78–80 ^ mechanisms require clarifying and intervention studies are at an early stage. Prolonged exposure and eye movement desensitization and reprogramming methods are currently being trialled for voice hearers with comorbid trauma.
Interpersonal relationship between hearer and voices	Methods explicitly described by Hayward et al^ 61,81 ^. Also a significant element of applying computerized avatar representations to voices^ 62 ^ and voice dialog.^ 64 ^	Evidence that voices can be understood within interpersonal frameworks and that this can predict distress.^ 82 ^ Therapeutic value of targeting relationship with voices is supported by avatar therapy trial.^ 62 ^ A pilot RCT of relationally based CBT is underway.

*Note:* ACT, acceptance and commitment therapy; CBTp, cognitive behavioral therapy for psychosis; CMT, compassionate mind training; COMET, competitive memory training; RCT, randomized controlled trail; TAU, treatment as usual.

### Targeting Psychological Processes Associated With Voices

In considering how to focus more on therapeutic processes, it is notable that the therapy approaches developed for voices have mainly been derived from extension of therapy methods developed for anxiety and depressive disorders to voices (eg, cognitive therapy, mindfulness, CMT) rather than being driven by research on processes involved in voices themselves, which may better guide therapy.

In considering this, one key area in need of development is to establish methods for addressing the observed relationship between trauma and voices.^73,74,78–80^ A recent meta-analysis has shown that childhood abuse is associated with adult psychotic disorder with an estimated population attributed fraction of 33%,^83^ and different childhood adversities are associated with different symptoms, with sexual abuse specifically associated with auditory hallucinations.^80^ Paralleling this, voices are common in people with posttraumatic stress disorder (PTSD).^84^ The potential role of cognitive factors, such as negative self/other evaluations due to traumatic experiences, and responses to trauma, such as dissociation, is ripe for research.^85,86^ However, the literature is at an early stage in operationalizing how to work with trauma when it is associated with voices. Approaches to date have mainly focused on incorporating past experiences into shared formulation,^18,63^ rather than directly targeting trauma-related memories or processes involved in their manifestation in voice experience. Methods such as cognitive restructuring of the meaning of trauma memories, imagery rescripting, prolonged exposure, and eye movement desensitization and reprocessing are beginning to be studied in people with comorbid PTSD and psychosis.^75–77,87^ This work is important in informing methods of working with the link between trauma and voices specifically. Given proposals that trauma-related dissociative tendencies might confer a vulnerability to voices,^88^ other areas of potential development might include the application of therapeutic techniques informed by cognitive models of dissociation.^89^

Second, there appears a need to consider the cognitive mechanisms involved with voices. With delusions, there has been recent progress in moving beyond adaptations of standard cognitive restructuring to develop therapy approaches that more explicitly target cognitive processes specifically associated with delusions, such as reasoning biases.^90,91^ However, therapy methods have yet to be developed to target underlying neurocognitive mechanisms specifically associated with voices, such as source-monitoring and cognitive control difficulties.^92^ Indeed, therapy has developed primarily with the aims of promoting adaptation to voice experience, rather than attempting to help voice hearers to reduce the occurrence of their voices. Early attempts to overcome source-monitoring difficulties using interventions which helped people focus on their voices^36^ did not progress. As neurocognitive models of voices extend beyond simple models of a core source-monitoring problem, broader cognitive targets may become viable. For example, considering the relation of some voices to previous aversive events and evidence from neuroimaging studies of involvement of the parahippocampal gyrus during auditory hallucinations,^93^ a significant number of voices appear to have memory processes implicated in them. Furthermore, there is mounting evidence that social cognition may be important in the experience of voices,^94^ and consequently therapies emphasizing interpersonal aspects are likely to be promising. As these mechanisms become better conceptualized interventions may become clearer.

Third, it is notable that the roots of psychological interventions in cognitive therapy approaches for emotional disorders has meant that therapeutic outcomes for voices have been studied most widely in relation to the emotional consequences of hearing voices (ie, voice-related distress and depression), rather than broader processes of individual adaptation and personal recovery. Processes highlighted by service users as central to personal recovery in psychosis include: developing hope; empowerment and responsibility in self-management of problems; developing a view of self beyond the stigmatized and subjugated role of psychiatric patient; finding new life directions; and social connection.^95^ While these targets are often incorporated into working formulations by practitioners, the operationalization of methods for working with these has been less well developed and evaluated.

### Focused Measurement of Outcome

In addition to clarifying the specific methods and psychological processes to be addressed by therapies for voices, there is a need to tighten conceptualization and measurement of outcome. Psychological therapy trials for psychosis have been criticized for using outcome measures of symptom intensity used in antipsychotic drug trials, not well suited to the aims of psychological therapies of reducing impacts of symptoms on emotional well-being and functioning rather than their occurrence.^96^ The main voice-specific outcome measure used has been the Psychotic Symptom Rating Scales (PSYRATS)^97^ (see Woodward et al, this issue). Although capturing a number of dimensions, this measure has limitations in giving a total score which sums a series of only modestly intercorrelated scales, and which introduces noise from variables not targeted by therapy (such as voice frequency, location, and extent of negative voice content), while individual items are comprised of single 5-point ratings, likely to lack sensitivity to change when used instead. Indeed, different methods of scoring have been used between trials (single item, factor, or total score), reducing comparability of findings. Better measures are needed to capture the impact of voices on emotional well-being and functioning. Multiple item measures of the subjective impact of psychosis have been developed in recent years,^98,99^ which are likely to be more sensitive, but have yet to be reported on in trials, and are not voice specific. The application of these measures, and development of a voice-specific measure, are required to provide a more accurate estimate of targeted voice outcomes posttreatment and at follow-up.

This needs to be conducted in conjunction with more attention to measuring psychological processes hypothesized to mediate outcomes. This would include, eg, measuring beliefs about voice power and intent, acceptance of voices, and the capacity to decenter from voices. Novel instrumental variable methods, which have been developed specifically for the purpose of analyzing data from psychotherapy trials, can be used to show whether mediators of interest are causal in bringing about a therapeutic outcome.^100^

### Understanding Individual Differences

In considering the potential range of therapeutic targets that are emerging, an important issue is to improve ways of conceptualizing individual differences in hearing voices. CBT-based approaches have evolved to be heavily focused on individualized case conceptualization as the key means of determining therapeutic direction, conducted using basic transdiagnostic principles of functional analysis^13^ or cognitive formulation.^18^ This process would benefit significantly from research-based principles to aid in conceptualizing individual differences related to voice experience and prioritizing therapy targets.

A key issue on which participants may vary is the extent to which maladaptive beliefs about voice identity and power predominate, which may apply to only a proportion of people.^81^ Potentially this may be most applicable to persons with command hallucinations or threatening voice content, especially those who easily become drawn in to regarding their voices as sentient others. In others the focus may be on derogatory voice content, which may reflect negative self-schemas, or on the intrusiveness of the experience, which may require alternative methods, such as COMET.^84^

It has also been argued that patients may present with distinct subtypes of voices, with each having different causes and requiring different forms of treatment (see McCarthy-Jones et al, this issue). For example, a subtype called hypervigilance auditory hallucination has been described in which there is an exaggeration of the normally adaptive perceptual bias humans evolved to detect threat resulting in auditory “false-positives” from the environment which confirm beliefs regarding feared public exposure of shaming information.^101,102^ Mechanisms here seem distinct from intrusions of self-critical or trauma-related cognition into consciousness. Further development of such clusterings of voice experience and mechanisms is a potential direction in identifying ways of conceptualizing individual differences.

In examining the applicability of different methods to different individuals, as well as conducting dedicated trials of specific methods there may be value in considering alternative methods of data analysis. Clinical trials of psychosocial interventions typically compare the average outcomes of individuals randomly assigned to different treatment conditions. However, because no treatment is equally effective for all recipients, the average response, as measured, eg, by clinical rating scales, is less important than determining which participants have a response. In the case of antipsychotics, eg, the average response in comparison with placebo is often statistically significant but masks individual variability in response.^103^ New statistical techniques have enabled researchers to isolate the proportion of patients (about 20%) that show a dramatic and rapid improvement in their positive symptoms.^104^ These growth mixture modeling or latent trajectory analyses have obvious applications in psychosocial trials and may facilitate the identification of subgroups of voice hearers who are responsive (and nonresponsive) to different psychological therapies.

### Extending Beyond Schizophrenia and Beyond Voices

It is similarly important to note that voices span diagnostic categories, yet therapies have been developed primarily for persons with a diagnosis of schizophrenia. Auditory hallucinations have been documented in PTSD with a prevalence of 40%–50%; 37% in bipolar 1 disorder, 30% in borderline personality disorder; 10%–23% in major depression; 14% in obsessive compulsive disorder; 14% in dementia; and 10% in Parkinson disease.^84^ The focus to date on schizophrenia spectrum disorders likely reflects these being the diagnoses in which they are regarded a primary symptom, rather than secondary or comorbid feature, and hence a priority treatment target. However, there remains a need to consider how to intervene with voices in the context of other presentations. It is notable that voice characteristics can be broadly similar across different diagnostic groups.^105^ However, assumptions that methods will generalize to non-schizophrenia populations, such as those with borderline personality or dissociative disorder diagnoses, are yet to be tested.

Similarly, voice experience needs to be considered in conjunction with the co-occurrence of hallucinatory experiences in other sensory modalities, which have been neglected within the therapy literature until only very recently.

### Shaping Interventions for Service Implementation

Finally there needs to be consideration of the evolving service contexts for the delivery of psychological therapies for voices. In the United Kingdom, dissemination of psychological therapies for psychosis has been supported by leadership from pioneers of therapies for psychosis and by prioritization in the National Institute for Clinical Excellence guidelines.^4^ However, there have been significant challenges,^106^ and implementation has been limited in other countries.^9^ While this may in part reflect conflicting service priorities, it should be acknowledged that therapeutic approaches for voice hearers described to date usually require advanced levels of therapy skill, which is a barrier to widespread dissemination. In addition there may be variable levels of client demand for formal psychological therapies. In the context of these barriers, it may be important to shape interventions suitable for routine delivery in service contexts, which may require going beyond the traditional model of consultation room delivery by an expert therapist.

Major developments which may influence this include the increasing use of online and mobile technology as media for delivering interventions, increasing employment of graduate level mental health workers, and an increasing role of peers with lived experience in provision of services. These developments each demand the availability of simpler, or lower intensity, intervention models, which may benefit from the development of simple focused intervention methods aligned with particular therapeutic targets and processes (see Hayward et al^107^ for an example of a self-help intervention).

While challenges have been apparent in conducting individualized cognitive restructuring in a group setting, and early group CBTp trials had relatively disappointing results on voice and positive symptom outcomes per se,^49,50,108^ more recent therapy developments such as mindfulness-based therapies potentially have better fit with a group format. Hence use of groups which combine sharing lived experience alongside structured therapy methods may be a means of integrating targeted therapeutic methods with peer support which has long been proposed by voice hearers as facilitative of personal recovery processes (see Corstens et al, this issue). Meanwhile, new methods of delivery present opportunities, including the potential for peer delivery and online media to facilitate modeling from others with lived experience; for the therapeutic use of technology such as avatar representations of voices; and for social networking to promote belonging among persons with a private and difficult to understand experience.

## Conclusion

RCT data for the use of CBTp in schizophrenia supports the idea that including psychological therapy in addition to routine care is more beneficial than routine care alone on participants’ report of psychotic symptom severity, with very recent data suggesting that this extends to measures of overall voice severity. However, closer examination of the data relating specifically to voices suggests that our understanding of which specific methods are useful for promoting expected outcomes for voices as a specific treatment target remains limited. To address this, we may need to graduate from the focus on whether broad therapy approaches such as CBTp demonstrate effects on generic symptom outcomes onto more targeted research to better understand specific processes, therapeutic methods, and applicability for different voice hearers and service delivery contexts.

## Funding

S.M.-J.’s contribution to this research was supported by a Macquarie University Fellowship and Wellcome Trust Award 098455/Z/12/Z.
